# Evaluation de la satisfaction des patientes hospitalisées au service de gynécologie obstétrique de Sousse, Tunisie

**DOI:** 10.4314/pamj.v8i1.71161

**Published:** 2011-04-18

**Authors:** Iheb Bougmiza, Meriem EL Ghardallou, Chekib Zedini, Hatem Lahouimel, Thouraya Nabli-Ajmi, Ridha Gataa, Imen Touati, Hedi Khairi, Ali Mtiraoui

**Affiliations:** 1 Département de Médecine Communautaire. Faculté de Médecine Ibn El Jazzar, Université de Sousse, Tunisie; 2 Service de gynécologie obstétrique, hôpital Farhat Hached, Sousse, Tunisie; 3 Unité de recherche Pratique Médicale Ambulatoire PMA 01/UR/ 08-15, Tunisie; 4 Laboratoire de recherche Qualité des Soins et Management des Services de Santé Maternelle, Tunisie

**Keywords:** Qualité des soins, satisfaction des patients, gynécologie, Tunisie

## Abstract

**Introduction:**

Il est admis que la satisfaction des patients est un indicateur de la qualité des soins. L’objectif de ce travail était de mesurer la satisfaction des patientes du service de gynécologie obstétrique du CHU Farhat Hached de Sousse.

**Méthodes:**

Une étude transversale a été menée entre mai et octobre 2005. La collecte des données a été faite par trois internes grâce à des entrevues structurées avec des patientes et à partir d’un questionnaire validé.

**Résultats:**

600 patientes ont été interviewées. L’âge médian était de 30 ans. Prés de quatre vingt cinq pour cent des femmes étaient instruites. Les patientes analphabètes ont exprimé une meilleure satisfaction (score de 56,6 %) par rapport aux autres (p < 10^-3^). Par ailleurs, nous avons noté : un niveau de satisfaction globale moyen (score de 51%); un faible niveau de satisfaction pour la restauration et les conditions de séjour; un bon niveau de satisfaction pour les soins médicaux et paramédicaux. Les patientes les plus satisfaites étaient celles qui avaient l’impression que leur état de santé s’est amélioré, celles qui s’attendaient à des services de qualité moindre ou de qualité similaire, et celles qui avaient l’intention de recommander l’hôpital à leurs proches.

**Conclusion:**

Il est difficile de transposer les résultats de travaux étrangers au contexte tunisien, en raison des différences entre les systèmes de santé d’une part et les caractéristiques socio-démographiques des patientes, d’autre part. En revanche, certains domaines devraient être examinés par des professionnels de la santé afin d’assurer la qualité requise.

## Introduction

L’intérêt porté à la qualité des soins n’a cessé de croître ces dernières années. La majorité des établissements de santé la placent au coeur de leurs priorités. Les raisons en sont multiples A savoir, d’une part, l’évolution constante et rapide de la médecine grâce aux progrès scientifiques, et d’autre part, les patientes ont des revendications de plus en plus nombreuses concernant l’accessibilité aux soins et aux nouvelles technologies, tout en exigeant des garanties de sécurité [1]. Les patients remettent en question les décisions de leur médecin, les changent s’ils ne sont pas satisfaits. Ils exigent des résultats et forment même des associations pour défendre leurs intérêts. Bref, ils se comportent en clients.

Ainsi, l’effort est lancé afin de répondre non seulement aux normes techniques d’un service, mais aussi pour satisfaire les attentes des clients les plus variées. On ne parle plus de qualité technique uniquement, mais aussi de qualité perçue.

La mesure de la qualité des soins d’une hospitalisation doit certes, tenir compte du point de vue des acteurs de soins, mais aussi de celui du patient [1,2].

La manifestation verbale ou écrite de la satisfaction du patient est un jugement qu’il porte sur tous les aspects des soins, et particulièrement la dimension inter-personnelle. Certains patients sont reconnaissants alors que la qualité des soins n’était pas optimale. Inversement, il existe des patients pour lesquels les conditions hospitalières ainsi que celles des soins fournis ont été les meilleures, mais qui se plaignent de leur sort.

La satisfaction est ainsi, une grandeur subjective qui reflète les préférences et les attentes personnelles des patients. Les perceptions individuelles, forcément subjectives, d’une réalité vécue par les patients, peuvent être différentes de la réalité objective de l’expérience d’hospitalisation, et ne pas correspondre à celles des personnels de soin et des gestionnaires [3-6].

La satisfaction des patients est considérée comme un indicateur de la qualité des soins [7,8]. Elle est corrélée à l’adhésion thérapeutique, à la continuité des soins et à l’amélioration de l’état de santé tel qu’il est perçu par le patient [9].

La mesure de la satisfaction des patients se fait généralement à l’aide d’un questionnaire qui explore ses multiples dimensions. A chaque enquête correspond un questionnaire différent, ce qui permet d’aborder chaque problème dans son contexte [7].

Ce travail entre dans le cadre de la stratégie nationale d’amélioration continue de la qualité des soins et des services de santé en Tunisie.

Nos objectifs étaient de mesurer la satisfaction globale et spécifique des patientes hospitalisées dans le service de gynécologie obstétrique de Sousse, et d’en analyser le lien avec leurs caractéristiques sociodémographiques.

## Méthodes

**Type et population d’étude**

Nous avons opté pour une étude transversale menée auprès des patientes hospitalisées au service de gynécologie-obstétrique du CHU Farhat Hached de Sousse (Tunisie), durant la période allant de Mai à Octobre 2005.

Ont été incluses toutes les patientes admises, pendant la période de l’étude, dans trois unités du service : l’unité des grossesses à risque, l’unité de gynécologie et l’unité du post partum. N’ont pas été incluses les femmes âgées de moins de 15 ans, les femmes qui ont présenté un problème de communication (sourde muette, barrière linguistique…) et celles qui avaient une détérioration mentale ou une pathologie tumorale à un stade avancé. Si la patiente avait été hospitalisée plus d’une fois durant la période de l’étude, elle n’était interviewée qu’une seule fois.

L’estimation de la taille de l’échantillon a été faite en considérant un taux de satisfaction globale de 70 % avec une pondération pour les types d’unités. Nous avons supposé qu’il n’y avait pas de variation temporelle de la satisfaction pendant la période de l’étude. Afin de pouvoir faire une analyse efficace par unité, un effectif minimum de 100 dans chaque unité était nécessaire [10].

**Recueil des données**

Nous nous sommes basés sur un questionnaire qui a été testé dans un travail antérieur, mené dans un autre hôpital tunisien (Kairouan) [11]. Le questionnaire a été écrit et administré en arabe. Il comprenait 57 items explorant les différentes dimensions de la satisfaction : la satisfaction globale (1 item), la satisfaction spécifique quant à la prise en charge aux urgences (4 items), la satisfaction spécifique concernant les modalités d’admission (3 items), le temps pour avoir un rendez-vous d’hospitalisation (1 item), la satisfaction spécifique concernant les services de restauration (5 items), la satisfaction spécifique concernant l’environnement et les conditions de séjour (8 items), la satisfaction spécifique concernant les soins infirmiers (12 items) et la satisfaction spécifique concernant les soins médicaux (9 items). L’intention de recommander l’établissement à un proche, ainsi que de les données sociodémographiques des patientes (9 items) et médico-administratives (5 items) ont été aussi relevés.

Pour chaque item, la patiente était invitée à répondre sur une échelle ordinale de Likert en 4 points : excellent, bon, faible, et très faible, qui étaient codés respectivement de 3 à 0.

Le questionnaire renfermait des items sur les attentes des patientes envers les services reçus avant et après l’hospitalisation, ainsi que des items sur l’évaluation des résultats de l’hospitalisation en matière d’amélioration de l’état de santé.

La collecte des données a été réalisée par trois internes en médecine générale préalablement formés à la technique d’entrevue.

Ces entrevues ont été réalisées directement auprès des patientes hospitalisées en fin de matinée, quand les patientes étaient déclarées sortantes et avant que les formalités administratives ne soient entamées.

Le consentement des patientes ou de leurs proches était recueilli avant chaque entretien moyennant une information courtoise relative aux objectifs du travail.

Ce travail a fait l’objet d’une thèse de doctorat en médecine soutenue en 2006 à la faculté de médecine de Sousse (Tunisie).

**Analyse statistique des données**

Nous avons testé la normalité des variables quantitatives à l’aide du test de Kolmogorov Smirnov. Quand la distribution était normale, ces variables étaient représentées par la moyenne avec son écart type, le cas échéant nous avons utilisé la médiane avec les extrêmes.

Nous avons calculé des fréquences simples des scores moyens et des pourcentages scores pour chaque item, en accordant une pondération différente en fonction de la réponse (voir la formule ci-dessous).

Score moyen = (3×n excellent) + (2×n bon) + (1×n faible)/N

Le pourcentage score était obtenu en divisant le score moyen par 3 et en le multipliant par 100. Lorsque le pourcentage score était inférieur à 50 %, la variable étudiée était jugée comme insatisfaisante par les patientes.

Nous nous sommes aussi intéressés à l’analyse des déterminants de la satisfaction globale en menant des analyses bivariées. Le test de Chi deux a été utilisé pour la comparaison des pourcentages.

La saisie et l’analyse des données ont été confiées à une structure universitaire externe à l’établissement afin de garantir un maximum de neutralité. Elles ont été effectuées sur Epi-Info 6.04. Le risque d’erreur consenti était de 5 %.

## Résultats

**Description de la population étudiée**

Au total 600 femmes, réparties à part égale dans l’unité de gynécologie, l’unité des grossesses à risque et l’unité des femmes accouchées, ont été interviewées. L’âge médian des femmes interrogées était de 30 ans, avec des extrêmes allant de 16 à 86 ans.

La majorité des femmes enquêtées étaient instruites (84,5 %). Les patientes analphabètes ont exprimé une meilleure satisfaction (score = 56,6 %). Le pourcentage score décroît à mesure que le niveau d’instruction s’améliore (p < 10-3) (Tableau 1).

Quatre cent soixante trois patientes (77,1 %) étaient couvertes par un régime d’assurance maladie. Quatre cent vingt patientes (70 %) avaient un faible revenu puisqu’elles gagnaient moins de 200 Dinars (144,7 USD) par mois correspondant eu SMIG en Tunisie. 72,0 % des patientes (n = 432) étaient admises par le biais des urgences. 25,0 % étaient admises par le biais des consultations externes et 2,8 % avaient été transférées d’un autre service. La durée médiane d’hospitalisation était de 3 jours et variait entre 1 et 47 jours.

**Evaluation des résultats de l’hospitalisation**

57,3% des patientes ont perçu une grande amélioration de leur état de santé en fin d’hospitalisation. Les patientes de l’unité des grossesses à risque ont le plus perçu cette amélioration avec un pourcentage de 62,0 %.

Dans l’ensemble, les prestations fournies correspondaient aux attentes des patientes. Celles qui s’attendaient à de meilleurs services ont représenté 35,1 % de l’ensemble des patientes interviewées. Par ailleurs, 70,1 % des patientes recommanderaient à leurs proches de se faire soigner dans ce service si leur état le nécessite (Tableau 2).

**Evaluation de la satisfaction globale des soins**

La satisfaction globale des patientes était moyenne. Le score moyen était de 1,53 avec un pourcentage score de 51%. Nous avons noté une meilleure satisfaction des patientes admises dans l’unité des grossesses à risque, par rapport aux autres unités, avec un score de 54,6 %. (p = 0, 02)

**Evaluation des dimensions spécifiques de la satisfaction**

**Les urgences**

L’évaluation de la satisfaction des patientes de l’unité des urgences gynéco-obstétriques n’a concerné que les femmes admises par le biais des urgences (n = 444). Le temps d’attente médian avant de voir le médecin était de 10 minutes avec des extrêmes allant de 1 à 360 minutes. Concernant le temps total passé aux urgences, la médiane était à 45 minutes. Il variait entre 1 et 660 minutes. Les pourcentages scores relatifs aux deux items précédents, ainsi qu’à l’attention du staff des urgences et les explications fournies, étaient tous supérieurs à 50 % (Tableau 3).

**L’admission**

496 patientes n’ont pas répondu aux questions relatives à l’évaluation des procédures d’admission puisque ces dernières ont été accomplies par leurs accompagnants. Le temps médian pour accomplir les procédures nécessaires était de 60 minutes. Ce temps variait entre 1 et 240 minutes. L’évaluation de la satisfaction des patientes concernant cet item ainsi que la courtoisie du staff, la propreté et le confort de la salle d’attente, était jugée bonne dans l’ensemble, comme en témoignent les pourcentages scores obtenus (Tableau 3)

**Les conditions de séjour**

L’évaluation des conditions de séjour et de l’environnement physique des patientes était globalement négative. Ceci a concerné principalement la propreté des blocs sanitaires avec un pourcentage score très faible à 11,7 %. Le niveau de bruit diurne était, lui aussi, à l’origine d’une grande insatisfaction. (score = 37,6 %). Concernant le confort du lit, les patientes étaient insatisfaites (score = 44,4 %) surtout pour les femmes accouchées qui trouvaient que leurs lits étaient étroits pour elles et pour leurs bébés (Tableau 3).

**La restauration**

50,6 % des patientes ont fourni leurs appréciations au sujet de la restauration. Le reste des patientes ont consommé des repas préparés par leurs familles. Globalement, les patientes étaient insatisfaites concernant la température, le goût des aliments ainsi que l’horaire de passage du service des repas. En effet, les aliments étaient souvent froid, pas bon et servis en retard (Tableau 3).

**Les soins médicaux et paramédicaux**

Les aspects qui n’ont pas suscité la satisfaction des patientes ont concerné la communication, de façon générale, avec le personnel paramédical (pourcentage score de 37,4 %) ainsi que les explications fournies quand elles sont demandées (pourcentage score de 42, 7 %). Le pourcentage score le plus bas enregistré (35,4 %) a concerné l’assistance des infirmiers pour les gestes de la vie quotidienne (Faire manger les patientes, les amener à la salle de bain…).

L’évaluation des soins médicaux étaititle généralement bonne. En effet, les patientes trouvent que les médecins sont compétents, courtois et respectueux. Les pourcentages scores les plus bas étaient notés pour la disponibilité des médecins (54,6 %) et les instructions données aux patientes à la sortie de l’hôpital (54,5 %) (Tableau 4).

## Discussion

**Considérations méthodologiques**

La littérature internationale sur les enquêtes de mesure de la satisfaction, majoritairement anglosaxonne, est abondante. D’une manière générale, les auteurs s’accordent sur le fait qu’à chaque enquête, correspond un questionnaire différent. Cette pratique a l’avantage de pouvoir aborder chaque problème dans son contexte, mais interdit toute comparaison et toute transposition directe des questionnaires déjà élaborés. Ceci est lié principalement aux différences existant entre les caractéristiques des populations étudiées, et entre les organisations des systèmes de soins [7]. Certains auteurs parlent même de la nécessité d’une validation culturelle des résultats [12,13].

Le questionnaire a été conçu et administré en arabe. Cependant, quelques questions pouvaient être mal interprétées ou mal comprises. Pour palier à ce problème, un pré test a été effectué sur un nombre réduit de patientes.

Nous avons opté pour une interview en face à face puisque 14 % de nos patientes étaient illettrées. Or, si ces illettrés représentent une minorité dans notre population d’étude, ce sont justement ces personnes issues de couches défavorisées, qui sont susceptibles de présenter différents problèmes liés à la communication, à l’accès aux soins et donc présenter des spécificités en termes de satisfaction.

L’interview en face à face offre l’avantage de réduire le taux de non réponse et de données manquantes, par comparaison au questionnaire adressé par voie postale ou par contact téléphonique qui coûte plus cher. De plus, il permet de standardiser les conditions de remplissage. Il a par contre l’inconvénient de ne pas respecter l’anonymat et d’exposer au biais de désirabilité sociale qui pourrait surestimer la satisfaction. Ainsi, le patient exprimerait moins facilement son insatisfaction [13,14].

Le questionnaire a été administré le jour de la sortie de l’hôpital malgré le fait que les patients expriment plus leur insatisfaction quand ils rentrent chez eux comme ils sont moins dépendants de leurs prestataires de soins [15-17]. Ce choix nous a été imposé en raison de la difficulté de contacter les patientes sortantes dont la majorité ne dispose pas de téléphone.

Quant à la nature des questions posées, elles étaient toutes fermées avec gradation. Ceci nous a facilité le traitement et l’analyse des données. Mais, nous a empêchés de valider certaines informations fournies et de tirer profit des avis des patientes, qui pouvaient découler de certaines questions ouvertes, pour améliorer la qualité de la prise en charge.

**Satisfaction globale**

Bien qu’il soit difficile de transposer les résultats des travaux étrangers au contexte Tunisien, le score de satisfaction globale enregistré dans notre étude est relativement bas (51%) par rapport à ce qui a été retrouvé ailleurs, où les scores variaient entre 71 % au Suède et 98, 7 % en Suisse [11,15,18-21].

Le faible score de satisfaction enregistré dans notre travail, peut s’expliquer premièrement par l’absence d’un système compétitif entre les établissements de santé Tunisiens, qui sont principalement publics. D’où l’intérêt d’appliquer le principe du benchmarking entre les établissements de santé, voire même entre les différents services d’un seul établissement. Ceci nécessite la standardisation de la méthodologie utilisée.

Deuxièmement, la rémunération des professionnels de la santé est indépendante du niveau de satisfaction des patients soignés. Troisièmement, le nombre important de patientes hospitalisées est responsable de la charge excessive de travail pour le personnel soignant, ce qui a un effet négatif sur la qualité des soins [22].

Il est communément admis que les variables sociodémographiques (âge, sexe, origine…) sont liées à la satisfaction globale [15,23,24]. Dans notre travail, seul le faible niveau socio-économique avait une influence statistiquement significative sur la satisfaction globale.

Quant à la répartition des scores de satisfaction selon les services d’admission, nous avons noté un meilleur score chez les femmes admises au service de grossesse à risque (54,6 %). Ceci peut s’expliquer par les différences entre les trois groupes de femmes pour les variables sociodémographiques cités ci-dessus influençant la satisfaction des patientes [25].

**Résultats des soins et attentes des patients**

Les patientes ayant eu une amélioration de leur état de santé en fin d’hospitalisation sont plus satisfaites que celles qui n’en ont pas eu [12].

Plus l’écart entre les attentes et les performances constatées dans les structures de soins est important, plus l’insatisfaction des patientes est grande. Les attentes des patientes sont souvent supérieures chez les femmes ayant un niveau d’instruction élevé [26].

**Satisfaction spécifique**

Le temps d’attente: Plusieurs auteurs ont trouvé que le temps d’attente est un facteur d’insatisfaction très important [18, 27]. D’autres études en revanche, [28, 29] ont montré que la satisfaction des patients ne parait pas dépendre de la durée réelle de l’attente, du moment qu’elle correspond aux attentes des patients. Il faut ainsi se concenterr sur le changement de la perception du temps d’attente en informant les patients verbalement sur la durée d’attente probable, et ce dès leurs arrivée aux urgences, pour éviter toute confusion.

*La restauration et les conditions de séjour:* Les domaines dans lesquels un besoin d’amélioration se faisait plus sentir, ont concerné la restauration et les conditions de séjour. Concernant la satisfaction à l’égard des repas donnés, la quantité des aliments a figuré en tête de liste avec le meilleur score obtenu. Mais, ni la température, ni le goût et la variété des repas n’étaient satisfaisants. Ceci est lié probablement au fait que le service de restauration est laissé à des fournisseurs externes qui ne consultent pas les patients au sujet de leurs préférences alimentaires et par conséquent accordent peu d’importance à leurs satisfactions. Il convient de préciser que cette dimension prend beaucoup plus d’importance chez des malades hospitalisées pour de longues durées, contrairement à notre population pour qui la médiane de séjour était de trois jours.

Concernant les conditions de séjour, les dimensions recueillant les scores les plus bas ont concerné la qualité des blocs sanitaires, la température ambiante de la chambre ainsi que le niveau du bruit. Ceci implique la nécessité d’adapter l’infrastructure du service.

Les patientes se sont plaintes aussi de l’étroitesse des chambres et des difficultés de cohabitation dans un espace aussi restreint. En effet l’état des femmes varie en fonction du déroulement de l’accouchement et de l’état de santé du bébé.

*Les soins médicaux et paramédicaux:* Les soins paramédicaux avaient des pourcentages scores plutôt satisfaisants bien qu’on avait noté un déficit concernant l’assistance des patientes pour accomplir les gestes de la vie quotidienne. Ceci est lié principalement à la pénurie en aides-soignantes et à la charge excessive de travail.

Plusieurs patientes se sont plaintes du manque de disponibilité des médecins. Saultz a montré que la satisfaction augmente proportionnellement avec le temps de contact passé avec le médecin [30].

Un autre problème soulevé correspondant à une dimension cruciale pour tout patient, c’est le manque d’informations fournies. Ces informations n’ont pas concerné le séjour uniquement (planning des examens paracliniques, effets secondaires des médicaments pris…) mais aussi les informations données à la sortie.

A la lumière de ce dernier constat, et en l’absence d’un cadre légal qui régit l’information des patients, il paraît important de proposer aux professionnels de la santé davantage de séances de formation médicale continue en matière de communication soignant-soigné.

## Conclusion

Cette étude a permis de connaître les attentes prioritaires des patientes hospitalisées au service de gynécologie-obstétrique au CHU Farhat Hached de Sousse (Tunisie). Ces attentes constituent des thèmes privilégiés d’action pour améliorer la qualité des soins perçus. Bien qu’elle ne soit pas encore une mesure réglementaire en Tunisie, l’évaluation périodique de la satisfaction des patients doit être une procédure systématique pour les établissements de santé afin de se préparer à l’accréditation de nos institutions qui verra le jour dans un futur proche.

## Conflits d’intérêt

Ce travail a fait l’objet d’une thèse de doctorat en médecine, soutenue en 2006 à la faculté de médecine de Sousse (Tunisie). Autrement, les auteurs ne déclarent aucun conflit d’intérêt.

## Remerciements

Nos vifs remerciements s’adressent au corps médical, paramédical et administratif du service de gynécologie obstétrique de l’hôpital Farhat Hache de Sousse.

## Figures and Tables

**Tableau 1:** Caractéristiques de la population étudiée et leur distribution en fonction des scores moyens de la satisfaction globale

	Fréquence absolue (n)	Fréquence relative (%)	Score moyen	Pourcentage score	p
**Age (année)**					
< 25	151	25,2	1,55	51,8	NS
25 -35	293	48,9	1,49	49,8	
36-45	105	17,5	1,52	50,8	
46 -60	40	6,7	1,67	55,8	
> 60	10	1,7	2,00	66,7	
**Niveau d’instruction**					
Analphabète	93	15,5	1,69	56,6	<10^−3^
Primaire	238	39,7	1,58	52,9	
Secondaire	188	31,3	1,48	49,3	
Universitaire	81	13,5	1,32	44,0	
**Mode de couverture sociale**					
Oui	463	77,1	1,52	50,7	NS
Indigents	66	11,0	1,65	55,0	
Payants	40	6,7	1,52	50,8	
Autres	31	5,2	1,45	48,4	
**Revenu**					
Faible	420	70,0	1,57	52,5	NS
Moyen	121	20,2	1,49	49,8	
Bon	59	9,8	1,33	44,6	

**Tableau 2:** Evaluation de la perception des patientes concernant les résultats de l’hospitalisation

Dimensions explorées	Fréquence absolue (n)	Fréquence relative (%)	Score moyen	Pourcentage score
**Etat de santé (n= 600)**				
Grande amélioration	344	57,3	1,60	53,5
Amélioration relative	216	36,0	1,44	48,3
Pas d’amélioration	40	6,7	1,41	47,0
**Attentes (n= 590)**				
Meilleurs services	207	35,1	1,15	38,3
Services similaires	240	40,7	1,72	57,3
Services de moindre qualité	143	24,2	1,73	57,8
**Recommandations à un proche (n = 598)**				
Oui fortement	419	70,1	1,68	56,0
Peut être oui	92	15,4	1,41	47,1
Peut être non	24	4,0	0,95	31,9
Non sûrement	63	10,5	0,29	30,6

**Tableau 3:** Evaluation des services fournis aux urgences, lors de l’admission et au moment du séjour

Les variables	N	Très faible		Faible		Bon		Excellent		Score monyen	% score
		N	%	N	%	N	%	N	%		
**Les urgences**											
Temps d’attente du médecin	425	40	9,4	55	12,9	285	67,0	45	10,6	1,78	59,6
Temps total passé aux urgences	424	58	13,6	54	12,7	283	66,7	29	6,8	1,66	55,6
Attention du staff des urgences	421	30	7,1	78	18,5	281	66,7	32	7,6	1,74	58,3
Explication donnée aux patientes	424	81	19,1	42	9,9	280	66,0	21	4,9	1,56	52,3
**L’admission**											
Temps nécessaire pour compléter les procédures	485	29	5,9	46	9,4	381	78,5	29	5,9	1,84	61,5
Courtoisie du staff d’admission	104	6	5,7	16	15,4	66	63,5	16	15,4	1,88	62,8
Confort et propreté de l’espace d’attente	101	5	5,0	15	14,9	67	66,3	14	13,9	1,89	63,0
**Les conditions du séjour**											
Propreté de la chambre	599	80	13,4	176	29,4	325	54,3	18	3,0	1,47	48,9
Propreté des toilettes	588	307	52,2	112	19,0	165	28,1	4	0,7	0,35	11,7
Propreté des couvertures	593	166	28,0	126	21,2	293	49,4	8	1,3	1,24	41,3
Temps de nettoyage	585	48	8,2	58	9,9	471	80,5	8	1,4	1,75	58,3
Bruit pendant le jour	595	186	31,3	160	26,9	236	39,3	13	2,2	1,12	37,6
Bruit pendant la nuit	587	122	20,8	115	19,6	210	35,8	140	23,9	1,62	54,2
Confort du lit	600	138	23,0	127	21,2	333	55,5	2	0,3	1,33	44,4
Température de la chambre	600	287	47,8	135	22,5	176	29,3	2	0,3	0,82	27,4
**La restauration**											
Goût de la nourriture	300	90	30,0	86	28,7	122	40,7	2	0,7	1,12	37,3
Température des aliments	296	132	44,6	72	24,3	92	31,1	0	0,0	0,86	28,8
Quantité des aliments	310	58	18,7	41	13,2	200	64,5	11	3,5	1,52	50,9
Plateau	306	66	21,6	46	15,0	193	63,1	1	0,3	1,42	47,4
Horaire	307	84	27,4	38	12,4	184	59,9	1	0,3	1,33	44,4

**Tableau 4:** Appréciation des soins médicaux et paramédicaux par les patientes

Les variables à l’étude	N	Très faible		Faible		Bon		Excellent		Score moyen	% score
		N	%	N	%	N	%	N	%		
**Soins médicaux**											
Courtoisie du médecin	599	10	1,7	46	7,7	456	76,1	87	14,5	2,03	67,8
Respect du médecin	599	4	0,7	43	7,2	462	77,1	90	15,0	2,06	68,8
Précision de l’examen	598	25	4,2	26	4,3	455	76,1	92	15,4	2,02	67,5
Compétence du médecin	589	5	0,8	21	3,6	479	81,3	84	14,3	2,08	69,7
Fréquence des visites	595	30	5,0	131	22,0	375	63,0	59	9,9	1,79	59,3
Disponibilité du médecin	472	72	15,3	82	17,4	263	55,7	55	11,7	1,63	54,6
Respect de l’intimité	589	1	0,2	11	1,9	459	77,9	118	20,0	2,17	72,6
Explications données aux patients	590	80	13,6	56	9,5	375	63,6	79	13,4	1,76	58,9
Instructions données	599	112	18,7	56	9,3	369	61,6	62	10,4	1,63	54,5
**Soins paramédicaux**											
Courtoisie des infirmiers	600	31	5,2	135	22,5	383	63,8	51	8,5	1,75	58,5
Habilité des infirmiers	600	22	3,7	67	11,2	475	79,2	36	6,0	1,87	62,5
Surveillance des infirmiers	600	62	10,3	102	17,0	404	67,3	32	5,3	1,67	55,9
Assistance des infirmiers	578	247	42,7	60	10,4	259	44,8	12	2,1	1,06	35,4
Réponse aux appels	523	137	26,2	124	23,7	251	48,0	11	2,1	1,26	42,0
Réponse aux demandes	504	149	29,6	102	20,2	241	47,8	12	2,0	1,23	41,0
Soutien moral	599	86	14,4	105	17,5	381	63,6	27	4,5	1,58	52,7
Respect de l’intimité	595	20	3,4	45	7,6	428	71,9	102	17,1	2,02	67,6
Attention au corps du patient	596	102	17,1	58	9,7	346	58,1	90	15,1	1,71	57,0
Explications données aux patients	597	189	31,7	75	12,6	308	51,6	25	4,2	1,28	42,7
Exécution des ordres	563	18	3,2	43	7,6	437	77,6	65	11,5	1,97	65,8
Communication	598	203	33,9	131	21,9	252	42,1	12	2,0	1,12	37,4
